# A Long-Term Perspective of FAS

**Published:** 1994

**Authors:** Ann P. Streissguth

**Affiliations:** Ann P. Streissguth, Ph.D., is professor and director of the Fetal Alcohol and Drug Unit, Department of Psychiatry and Behavioral Sciences, University of Washington, Seattle, Washington

## Abstract

Few people in their adolescent or adult years are diagnosed as having FAS. Studies have shown that most FAS patients outgrow the characteristic FAS facies after puberty. However, they may still suffer from mental handicaps and have poor age-appropriate life skills. Further research is needed to understand these patients’ unique needs and to develop the most effective intervention strategies.

In the 20 years since fetal alcohol syndrome (FAS) was identified as a birth defect, relatively few studies have described adolescents and adults with this disability, although FAS is thought to be the leading known cause of mental retardation. Many practical problems exist in accruing a population of adolescent and adult patients with FAS and in following them systematically past childhood. Yet by examining the natural history of FAS, it should be possible to evaluate the long-term needs of these patients and to develop an appropriate set of lifetime interventions suitable to their disabilities.

Taking a historical perspective, this article reviews the pertinent literature, describing the findings of several long-term followup studies of children with FAS. It raises questions about the needs of adolescents and adults with FAS that should be addressed through future research and public policy.

## FAS: The Initial Research

**Figure 1:**
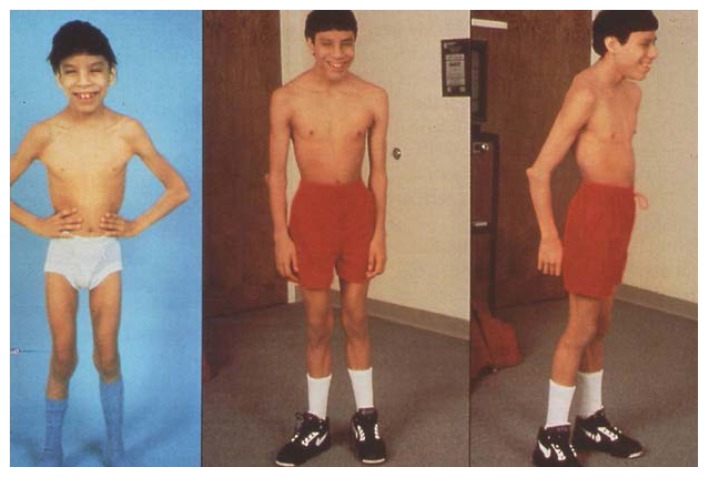
The first patient in whom FAS was diagnosed at birth at 8 and 18 years of age (also see figure 3A). SOURCE: Streissguth and Little 1994.

FAS is growing up. It is now 20 years since Jones and Smith (1973) coined the term “fetal alcohol syndrome” to describe children who had a specific and visually recognizable pattern of characteristics, and in July 1993, the first baby in the world to receive a diagnosis of FAS at birth celebrated his 20th birthday (figure 1). Children with similar characteristics had been independently identified in France (Lemoine et al. 1968) and in Seattle (Jones et al. 1973) as a specific subgroup of children born to alcoholic mothers.

**Figure 2:**
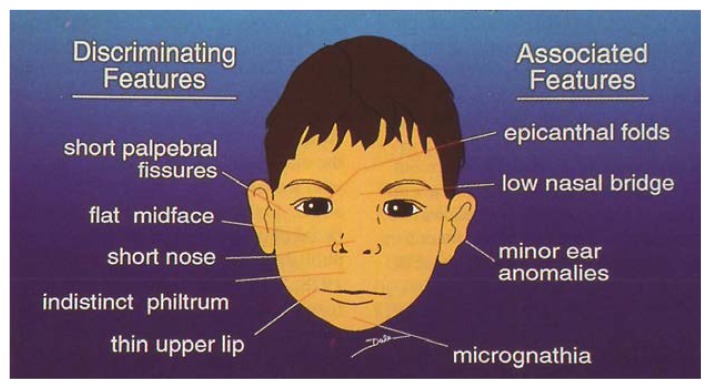
Facies in FAS particularly characteristic of the prepubertal child. Features on the left side are the most definitive. Those on the right side are less differentiating. Microcephaly (small head circumference) is not a facial feature per se, but a central nervous system characteristic. (Epicanthal folds: small fold of skin covering inner corner of the eye; philtrum: zone between nose and mouth; micrognathia: abnormal smallness of the jaws; palpebral fissures: eye openings.) SOURCE: Streissguth and Little 1994.

The characteristics of FAS were identified as growth deficiency; specific physical anomalies, including a characteristic facies (figure 2); and central nervous system (CNS) dysfunction. The CNS manifestations include delayed development, hyperactivity, motor incoordination, learning or attentional problems, seizures, mental retardation, and/or microcephaly (small head). These early insights on FAS suggested a biological cause for some of the problems seen in children of alcoholic^1^ mothers—and indicated that these problems could be prevented if women did not drink alcohol during pregnancy.

Two decades of laboratory research have validated these early findings on the causes of FAS. For example, experimental research has shown that alcohol is teratogenic^2^ across a wide variety of species and conditions of exposure, and it is the most commonly used known human teratogen in the world. Alcohol also causes FAS, with an estimated prevalence of 1–3/1,000 live births (National Institute on Alcohol Abuse and Alcoholism 1990).

### Early Research and Prevention Efforts

The early clinical reports of FAS by Lemoine and colleagues (1968) in western France and Jones and colleagues (1973) in Seattle were soon replicated by reports by Majewski and colleagues (1976) in western Germany, by Dehaene and colleagues (1977*a*) in northern France, and by Olegård and colleagues (1979) in Sweden. Like the initial reports, all of these studies in the 1970’s described patients with FAS who were infants and young children. Because these children with FAS were mostly functioning within the borderline and retarded range of intellectual development, FAS soon became recognized as one of the three most prevalent causes of mental retardation with a known etiology—and the only one that was entirely preventable.

This knowledge stimulated the development of important programs in the late 1970’s, designed to demonstrate methods to prevent FAS and to intervene in maternal alcohol abuse during pregnancy (Rosett et al. 1980; Rosett and Weiner 1981; Little and Streissguth 1981; Little et al. 1984, 1985). The United States Government initiated two important policies: an official government warning by the Surgeon General against drinking alcoholic beverages during pregnancy or when planning a pregnancy (Public Health Service 1981), and the labeling of alcoholic beverage containers with a warning about the risk of birth defects associated with drinking alcohol during pregnancy (Public Law 100–690, 1988). It is impossible to know how many children were helped as a result of these official policies, but it is probably a large number. Research projects such as those by Little and colleagues (1984), Rosett and colleagues (1980), and Larsson and colleagues (1985) have clearly demonstrated that curtailing alcohol use during pregnancy does improve pregnancy outcome.

Initially, researchers thought that the CNS effects of FAS were primarily manifested as mental retardation or microcephaly. There was an implicit assumption that the treatment and service needs of patients with FAS would be like those of patients with Down syndrome. (As a result of decades of research, a network of support services and special cradle-to-grave programs exists nationwide to accommodate the special needs of Down syndrome patients.) However, FAS did not turn out to be like Down syndrome. Children with FAS manifest a wide range of intellectual abilities, and no early biological marker has yet appeared to help identify those at risk. Clinicians who continued to work with patients with FAS as they grew up realized that they often behaved very differently than patients with Down syndrome, often getting into trouble in their communities, in their schools, and with their families. This awareness led us to the next set of research studies.

## Adolescent Manifestations of FAS

As early as the mid-1970’s, Smith made the diagnosis of FAS in two young men who were examined for the first time as adults; both were mentally retarded. Their facial photographs and IQ profiles were published in 1978 (Clarren and Smith 1978; Streissguth et al. 1978). In 1981, Iosub and colleagues described longitudinal data on three siblings with FAS who had been diagnosed in childhood. The two who had reached maturity had small heads, short palpebral fissures (eye openings), borderline to mild mental retardation, but variable growth deficiency; one was growth deficient only for height, the other only for weight.

During the first decade of FAS research, most clinicians seemed reluctant to make the initial diagnosis in adolescents and adults. This was understandable, as all the early patients identified were young children; the classic FAS face was the face of a young child (figure 2). Only patients with the most classic manifestations of FAS were identified initially as adolescents and adults. It was only when we were able to present systematic longitudinal data on the first children with a diagnosis of FAS that we were able to see how the physical features (the primary markers for the syndrome) changed with age, which makes initial identification more difficult in older patients. This first long-term FAS followup study (Streissguth et al. 1985) was a 10-year followup of the 11 children identified in Seattle in 1973. It revealed that the facial morphology of many persons with FAS changed as they matured. Longer noses and bigger chins often gave their faces a coarser look after puberty, a finding that has been confirmed by several other studies (Spohr et al. 1987, 1993; Lemoine and Lemoine 1992; Majewski 1993). Girls in particular tended to put on weight at this time, and their weight-to-height age ratio changed from low to high.

Does this mean that FAS attenuates in adolescents? Absolutely not. The decreasing specificity of the face and the growth deficiency after puberty only explains why initial identification of people with FAS after puberty can be more difficult. In FAS, the physical features are only the markers for the CNS deficits. As many studies of alcohol teratogenesis have demonstrated, the brain is the most vulnerable organ in the body to the effects of prenatal alcohol exposure (West 1986; Goodlett and West 1992; Streissguth et al. 1993). Although the physical features associated with FAS may change in adolescence, the CNS problems continue, often with more severe repercussions than those experienced in early childhood.

In this 10-year followup study (Streissguth et al. 1985), we also examined the intellectual and behavioral outcomes of these first children with FAS. Of the original 11, 2 had died, and 8 were available for followup. Four were functioning in the borderline-to-dull normal range of intelligence (IQ scores of 76 to 86), and four were clearly mentally retarded, with IQ scores of 20 to 57.^3^ The four who were mentally retarded were appropriately managed by schools and society, were in stable foster or adoptive situations, and were in appropriate programs for the mentally retarded, according to accepted guidelines (none were institutionalized).

The four who were *not* officially “mentally retarded” were the ones who seemed headed for trouble in the community, and subsequent observations confirmed this. One boy dropped out of school for the entire fifth grade year and only resumed school after moving to a different State. One girl dropped out of middle school and had a baby soon afterwards. The girl with the highest IQ and whose rearing background had seemed to be the most stable and supportive, ran away from home, lived a transient lifestyle, and became an unmarried teenage mother. The fourth has now been lost to followup. It is apparent that having a higher IQ did not assure these children a higher level of well-being; in a sense, it was not an “advantage.” This paradox motivated our next FAS followup study.

## Long-Term Consequences of FAS

Studies of adolescents and adults with FAS now have been conducted in several countries. The results show that FAS has lifelong consequences, with outcomes often more complex than those anticipated based on the fact that many patients with FAS are mentally retarded.

**Figure 3:**
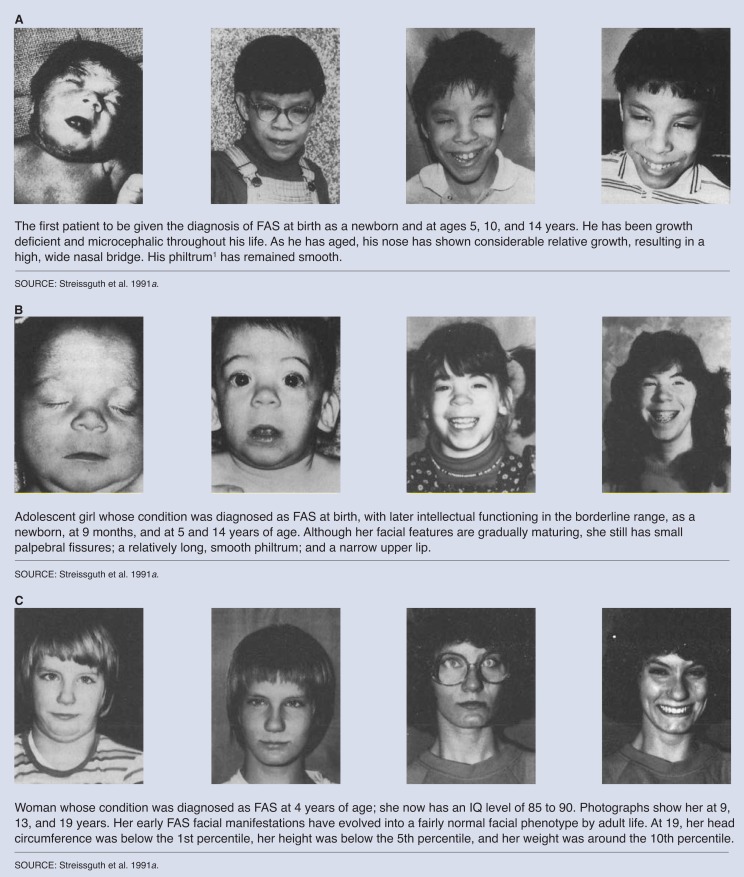
The evolving facial morphology of FAS patients, from childhood through adolescence. These changes can make it difficult to diagnose FAS in postpubescent patients. ^1^For definitions of these and other characteristics, see figure 2.

We published the first major databased study of the long-term consequences of FAS in adolescents and adults in 1991 (Streissguth et al. 1991*a*). This study, which involved 61 patients who ranged in age from 12 to 40 (mean age 17 years), confirmed that the physical features of FAS are less distinctive after puberty (figure 3). Four characteristics of the facial phenotype were noted: (1) continued growth of the nose in two dimensions (height of the nasal bridge and nasal length from root to tip); (2) continued growth of the midfacial region corrected the earlier midfacial hypoplasia; (3) improved soft-tissue modeling of the philtrum and upper lip; and (4) continued growth of the chin. In terms of growth deficiency, 25 percent of the adolescent and adult patients with FAS were not growth deficient for weight, and only 16 percent were not growth deficient for height; 28 percent were not microcephalic. Although the average IQ score of the adolescents and adults was 66, the range of intellectual development was broad; 42 percent had IQ scores not technically in the retarded range. This last finding has serious implications for the availability of appropriate services in schools and the community.

An additional study demonstrated general stability of IQ scores across a 5-year interval ending in adolescence or adulthood (Streissguth and Randels 1991*b*). Although these patients’ average academic functioning was at the second to fourth grade level, some did read and spell at a fifth grade level or beyond. A particular deficit in arithmetic skills was noted, which seemed related to difficulty with abstractions such as time and space, cause and effect, and generalizing from one situation to another. The severity of their arithmetic disability, often masked by superficial verbal skills, appeared to be central to their difficulty with independent living and poor judgment and their generally dysfunctional lives (Streissguth et al. 1991*a*).

In an attempt to document and quantify the type of adaptive living deficits noted clinically in our 1985 study, we used the Vineland Adaptive Behavior Scales (VABS) for the larger study of adolescents and adults. Although these patients had an average chronological age of 17 years, their overall level of adaptive functioning was at the 7-year-old level. Of the three domains measured by the VABS, the patients performed best on daily living skills (at an average 9-year-old level) and most poorly on socialization skills (at an average 6-year-old level). On average, these patients also showed significant difficulty with communication, as measured by the VABS. Only a few patients had age-appropriate daily living skills; none were age appropriate in terms of socialization or communication skills. Even patients with FAS or fetal alcohol effects (FAE) who were not technically retarded were frequently characterized on the VABS by such items as failing to consider consequences of their actions, lacking appropriate initiative, being unresponsive to subtle social cues, and lacking reciprocal friendships (Streissguth et al. 1991*a*).

In 1992, LaDue and colleagues published an additional report on this cohort, subsequently expanded to 92 patients, with a mean age of 18.4 years (age range 12 to 42). On a symptom checklist developed for this study, 80 percent of the patients were identified by their caretakers as having attentional problems, 73 percent had memory problems, and 72 percent were or had been hyperactive. On the VABS, 58 percent of the patients were identified as having maladaptive behavior scores in the “significant” range. This is a much higher proportion of patients with severe behavior problems compared with, for example, patients with Down syndrome (e.g., Harris 1988).

### Long-Term FAS Followup Studies From Europe

Reports from three sites in Germany and one in France have in general confirmed the findings of the Seattle studies. The long-term mental and behavioral consequences of FAS are serious, yet the patients are more difficult to recognize from their physical characteristics as they mature.

In 1992, Lemoine and Lemoine published an important study involving a 30-year followup of Lemoine’s original patients from Nantes, France. Many were found in state institutions as adults. Lemoine found that the facial dysmorphology changed radically in many patients, with the small nose and small chin of childhood giving way to a very elongated face with a large nose, a large chin, and coarse features. Lemoine had originally classified his patients in terms of severity of effects; those with the most severe growth deficiency and the most severe physical findings were called severe (75 percent of these patients had clear physical malformations). Others who had characteristic facial features and less severe growth deficiency were called mild (only 10 percent of these patients had physical malformations).

Lemoine found that severity of diagnosis was related to cause of death in patients with FAS. Among the 50 patients with severe FAS, 5 were known to have died, and 4 of these had died as children from severe cardiac problems or from a condition in which there is not enough oxygen in the body’s tissues (anoxia). Among the 28 patients with mild FAS in childhood, 2 had committed suicide as adults, and 5 others had attempted suicide.

Lemoine found that mental problems constituted the most severe manifestations of FAS in adulthood, including both intellectual retardation and behavioral problems. He described persistent behavior problems that prevented these patients from effectively using their intellectual potential and even their manual skills. He reported from his clinical observations that patients with FAS often could not focus on their work or their work environment because of their immaturity, considerable instability, and refusal to cooperate. Restlessness and hyperactivity concealed their lack of assurance and initiative and their need for assistance and protection. Although apparently euphoric and excited, they also were fearful, anxious, and depressed. Some were jokesters and comics; others were irritable and aggressive.

Lemoine also reported his observations on 16 additional former patients whom he had seen as children but had not classified as FAS. They had not had the characteristic physical features, although they had psychomotor retardation and alcoholic mothers. As adults, these patients also had significant behavior problems and were described as unable to stay focused on any activity. Six of these 16 patients were found in centers for the moderately disabled as adults. In the course of following up his former patients as adults, Lemoine also noted that 10 of the alcoholic mothers of the children he had diagnosed with FAS had undergone treatment for alcoholism and subsequently given birth to normal children who grew into normal adults.

Based on evaluations of his adult patients and on additional patients identified through the records as children of alcoholic mothers, Lemoine estimated that in five centers serving moderately disabled patients, 20 percent of the population were born to alcoholic mothers (25 of these 33 patients had severe FAS, 8 were without dysmorphic characteristics, and none were classified as mild FAS). In three institutions serving younger and less severely affected children and adolescents, he estimated that 14 percent of the population were born to alcoholic mothers (of these 42 patients, 6 were dysmorphic and 36 were not).

Lemoine concluded that there are severe long-term psychological problems associated with FAS that indicate the need for long-term professional services for these patients, even when they lack the characteristic dysmorphic features. These are the first known data that have attempted to address the prevalence of alcohol-related birth defects in institutions treating disabled persons.

Several studies from Germany also have described the lessening of the growth deficiency and the dysmorphic features associated with FAS as the patients mature physically (Spohr et al. 1987, 1993; Majewski 1993). Stability of IQ over time and the lack of general improvement in school status among adolescents also have been noted by Spohr in a study of 60 children with FAS who ranged in age from 8 to 15 years (1993).

In an important set of studies from Berlin, Steinhausen, a psychiatrist, and Spohr, a pediatrician, have been following a group of children with FAS as they grow older. In addition to confirming the diminished specificity of the facial features and growth deficiency with increasing age, these researchers also have documented the wide range of IQ scores, the general stability of IQ scores over time, and the general absence of change in IQ scores in an improved milieu (Spohr et al. 1987, 1993; Steinhausen et al. 1993, 1994).

What makes these Berlin studies unique is their attempt to quantify the behavioral disabilities often alluded to in the clinical descriptions of persons with FAS, particularly as they approach puberty and maturity. In two recent papers on the psychopathology of FAS, Steinhausen and colleagues (1993, 1994) used structured psychiatric interviews and the Achenbach Child Behavior Checklist, and accompanying Teacher Form, to examine profiles of psychiatric symptomatology. Changes in profiles of psychiatric symptomatology were examined across three ages: preschool, early school age (6 to 12 years), and late school age (13 years and older). Hyperkinetic disorders were the most frequent type of psychopathology at both the pre-school and the early school age period in a group of 27 children examined at both times. By the early school years, earlier problems such as enuresis (urinary incontinence) and eating disorders declined greatly, whereas speech disorders, emotional disorders, and unusual habits and stereotypic behaviors doubled in magnitude, characterizing approximately 45 to 50 percent of the sample.

Although unusual habits and stereotypic behaviors decreased by the late school years, emotional disorders, followed by hyperkinetic disorders, remained present in more than 50 percent of the 33 patients examined at both early and late school years. Conduct disorders remained constant in this group, at around 20 percent. Data from the Achenbach scales revealed attention-deficit problems as the most frequent problem, followed by social relationship problems, with the same pattern manifest at both examinations during the school age period. Both parent and teacher Achenbach scales revealed attentional and social problems to be the most prominent peaks in the symptom profiles. Although boys tended to have higher psychopathology scores than girls, there was little difference in the type of symptoms manifested in children with FAS within these age ranges.

## Future Directions for FAS Research

In the past few years, long-term followup studies of patients with FAS have been leading the field in a new direction. No longer can FAS be viewed as just a childhood disability—the changing needs of this population must be considered as they enter the adolescent years, which will (often for the first time) most clearly differentiate them from their peers. It is in early adolescence that many persons with FAS (even prior to a diagnosis) begin to express the feeling that they do not “think quite like everybody else.” It is at this age that parents may begin to realize that “just trying harder” is not the whole solution.

No longer can FAS be viewed as just another type of mental retardation. Not only are there many patients with FAS whose intellectual abilities fall well within the normal range, but they also are displaying an increasing and unsettling degree of recognizable psychopathology. Questions remain about the specific etiology of the psychopathology and behavioral disorders. Although it is always tempting to attribute psychopathology to adverse environments—and children with FAS have a greater likelihood of some adversity in their backgrounds—most followup studies of patients with FAS find a weaker than expected relationship.

Clinicians continue to see some patients with FAS who have severe cognitive deficits lasting into adulthood but who have not had adverse rearing environments. Clinicians also continue to see extremely variable outcomes in terms of secondary psychopathology and secondary disabilities that are not easily accounted for by either the level of the patients’ overall intellectual ability or the status of their rearing environment. These unsettling discrepancies demonstrate the need for more research: research on the affected patients themselves, research on their genetic predispositions, and research on effective ways to modify and improve behavioral outcome in individual patients.

A recent report of differential outcomes in dizygotic twins (fraternal twins) of alcoholic mothers should stimulate further investigation of the interaction of genetic and teratogenic influences on development (see Streissguth and Dehaene 1993). The almost complete absence of any literature on scientifically based interventions (either pharmacological or behavioral) should prompt researchers to study effective interventions for patients with FAS. It is hoped that the curious oversight by which FAS is not systematically addressed in the literature on children of alcoholics will be resolved so that more studies will focus (as Werner [1986] has done) on individual differences in children of alcoholic mothers and children of alcoholic fathers.

Documentation of prenatal alcohol exposure history should be a necessity in all studies of children of alcoholics, because both short-term (Day 1992) and long-term (Streissguth et al. 1994) consequences of prenatal alcohol exposure per se, even in the absence of maternal alcoholism or alcohol problems, has been reported from several cohort studies. Understanding the specific types of neuropsychological deficits that underlie FAS psychopathology will be an important challenge for the future, as will the complex interactions between individual levels of cognitive deficit and adverse and therapeutic environments.

The clinical work of the past 20 years also has suggested that what were thought to be suitable environmental interventions and educational opportunities for populations of retarded children (such as those with Down syndrome), are often not effective for patients with FAS. In particular, existing treatment and rehabilitative strategies seem less effective for people with FAS who are not classifiable as mentally retarded and for those whose mild cognitive deficits are compounded by attentional deficits or emotional instability.

Understanding the specific characteristics and needs of patients with FAS/FAE will permit development of the most appropriate interventions. Ideally such studies will not focus just on the preschool years and on academic attainment but will take a lifespan approach, with the goal of developing productive citizens who are capable of contributing to society at their own level of endowment. Recognizing the individual and specific needs of groups of patients with different types and etiologies of disabilities is an important current focus in developmental disabilities research (see Burack et al. 1988; Hodapp and Dykens 1991, 1992). This approach is needed for patients with FAS, who often have multidisciplinary problems that involve the home, the schools, the health care system, vocational training, the criminal justice system, and the community.

More and more, the behavioral characteristics of FAS appear broad and diverse rather than tightly constrained around a narrow cognitive dimension. Although some overlap between FAS and existing psychiatric nomenclature has been observed (see Shaywitz et al. 1980; Nanson 1992 and Nanson and Hiscock 1990 on attention deficits and childhood autism), FAS seems to have been largely ignored both in the psychiatric literature and in the psychiatric clinics in the United States. Steinhausen is the only psychiatrist who has published continuing followup studies (e.g., Steinhausen et al. 1993, 1994) that assess specific psychopathologic outcomes in patients with FAS. The recent study by Lemoine and Lemoine (1992) demonstrates the need for understanding prenatal alcohol exposure and FAS as a potential cause of psychiatric disability.

Diagnostic issues in the field are far from resolved. Not only does the diagnosis of FAS include recognizing a wide range of individual capabilities and differences, but there also are many fetal alcohol-affected children whose condition is not diagnosable because of the absence of specific facial characteristics. Without a diagnosis, such individuals often cannot receive the services they require.

As experimental animal studies accrue, an increasing body of research demonstrates both the early and the lifelong behavioral effects of alcohol in offspring who are neither growth deficient nor malformed (see Goodlett and West 1992; Riley 1990; Means et al. 1989; see the article by Becker et al., pp. 10–16). Even prospective longitudinal human cohort studies are showing a variety of behavioral problems at lower doses of alcohol than are necessary to produce physical effects (Day 1992; Streissguth et al. 1993). Further studies that emphasize the behavioral characteristics of alcohol teratogenesis are needed. By and large, it is the behavior and not the growth deficiency or anomalies that present the primary problems to the patients themselves, their families, and society.

The body of research reviewed here, all generated during the past 20 years about a developmental disability that previously had been unknown, is extensive. The research did not lead in the direction initially anticipated when FAS was first identified. Patients with FAS are not all mentally retarded, and their behavioral problems are often more debilitating than their cognitive deficits would suggest.

## Epilog

*One year ago I gave a public lecture on FAS. Seventy people attended. Afterward, three came up spontaneously to tell me their stories. Although the diagnostic speculations of these observers could not be confirmed, they illustrate the diverse faces of alcohol embryopathy that professionals see regularly in clinics serving patients with FAS/FAE. They also demonstrate the apparent relevance of the research on FAS to the individuals and families coping with related problems*.

*The first woman said, “My younger sister has FAS. I can tell from your pictures and she’s mentally retarded. I don’t have FAS,” she said, “I don’t look like those pictures and I’m not mentally retarded.” “But,” she told me, “I must have FAE. I’ve never been able to think like other kids and school was always very hard. My adoptive family had lots of testing done and sent me to psychiatrists, but they never figured out what was wrong with me. I just found out that my birth mother was an alcoholic. Now I’m 21, working on my GED, living on public assistance with my baby daughter—I still can’t figure things out. I don’t even know how to care for my daughter. Can you help me?” A man who approached me said his brother-in-law had FAE. He said his wife was the protective payee for her brother, who simply couldn’t manage his own affairs. Although the brother had attended school to become a chef, he couldn’t work as a chef; instead he is a kitchen aide, a job he is comfortable with due to its predictability and repetitive nature. He is surviving marginally in the community except when he drinks. His mother, who had a graduate degree and worked as a professional in the community, had had a serious drinking problem during the pregnancy with this young man but not during the pregnancy with her daughter. Now as adults, the daughter cares for the son*.

Finally, a sparkling 16-year-old girl spoke. “I’ve always been bright and a good learner and loved school,” she said, “but my mother has fetal alcohol syndrome. She looks just like those pictures and she’s quite unable to function as an adult. She’s always being victimized by men and really can’t take care of herself. She couldn’t take care of me either, that’s why I was adopted. I only found her recently. The thing I’m really thankful for,” she said, “is that she didn’t drink during her pregnancy with me.”

## Footnotes

1 The term “alcoholic” has been defined in many ways. Here the term includes “alcohol abuse” and/or binge consumption but does not necessarily imply dependence.

2 A teratogen is a substance that can affect a developing fetus, resulting in physical and neurological damage.

3 Most IQ tests have a mean of 100 (100 is considered normal) and a standard deviation of 15 points. An IQ of less than 70 is usually considered to reflect mental retardation, and IQ scores of 70 to 85 are considered to be in the borderline range.
